# Culture and personality disorders-a case series

**DOI:** 10.1192/j.eurpsy.2021.1801

**Published:** 2021-08-13

**Authors:** I. Cuevas Iñiguez, M.D.C. Molina Lietor, I. Moreno Alonso

**Affiliations:** 1 Psiquiatría, Hospital Universitario Príncipe de Asturias, Alcalá de Henares, Spain; 2 Psychiatry, Hospital Universitario Infanta Leonor, Madrid, Spain

**Keywords:** Culture, personality disorders

## Abstract

**Introduction:**

Personality disorders comprise a set of diagnosis characterized by inflexible, pervasive and enduring patterns of cognition, affect, behavioural and social interaction. The status of research on the personality among different cultures implies the universality of traits and disorders, as well as, their measuresacross cultures.

**Objectives:**

To study the prevalence of personality disorders in foreigner patients.

**Methods:**

In this retrospective case series, clinicaldata was collected from 40 patients who were hospitalized at the short-stay inpatient psychiatric service of the Príncipe de Asturias University Hospital during 2018.

**Results:**

Nineteen (47.5%) patients were European, ten (25%) were from South America, nine (22.5%) were Africanand two (5%) were Asian. Eight patients were diagnosed of diverse personality disorders. Seven (87.5%) of them were European, and only one (12.5%) was from South America.

**Conclusions:**

This case series suggests various directions for future research. The fact that patients diagnosed with personalitydisorders were mainly European could indicate diverse conclusions. It would question the universality of personality disorders out of a Euro-american frame of reference. It would also point out the difficulty of diagnosing personality disorders, taking into consideration language, awareness of cultural values,traditions, interactional patterns, and social norms. More studies of traits and personality are needed, taking into account the culture and the society in which patients have grown and in which they currently live.

**Disclosure:**

No significant relationships.

